# Implantation of a VDD implantable cardioverter-defibrillator lead via a persistent left superior vena cava

**DOI:** 10.1007/s00399-021-00835-7

**Published:** 2022-01-06

**Authors:** Mate Vamos, Laszlo Saghy, Gabor Bencsik

**Affiliations:** Cardiac Electrophysiology Division, Department of Internal Medicine, University of Szeged, Semmelweis u. 8., 6725 Szeged, Hungary

**Keywords:** LSVC, ICD, VDD, DX, Case report, LSVC, ICD, VDD, DX, Fallbericht

## Abstract

A persistent left superior vena cava (LSVC) represents a challenging congenital abnormality for transvenous cardiac device implantation. In the current case a secondary prophylactic VDD implantable cardioverter-defibrillator (ICD) implantation was planned in a 75-year-old woman presenting with ischemic cardiomyopathy and elevated stroke risk. Since no venous communication to the right side was identified intraoperatively, the lead was placed via the persistent LSVC. The far-field signal on the floating atrial dipole could be successfully blanked out, and appropriate device function with high and stable atrial sensing was demonstrated at follow-up.

## Abstract (de)

Eine persistierende linke obere Hohlvene ist eine angeborene Anomalie, die anspruchsvoll hinsichtlich der transvenösen Implantation eines kardialen Geräts sein kann. Bei einer 75-jährigen Patientin mit ischämischer Kardiomyopathie und erhöhtem Schlaganfallrisiko wurde eine sekundärprophylaktische VDD-ICD-Implantation geplant (*ICD* implantierbarer Kardioverter-Defibrillator). Da intraoperativ keine venöse Verbindung zur rechten Seite festzustellen war, wurde die Elektrode über die persistierende linke obere Hohlvene gelegt. Das Fernfeldsignal am flottierenden atrialen Dipol konnte erfolgreich ausgeblendet und eine adäquate Gerätefunktion mit guter und stabiler atrialer Wahrnehmung in der Nachsorge nachgewiesen werden.

## Medical history

The case of a 75-year-old woman with ventricular tachycardia accompanied by hemodynamic instability due to ischemic cardiomyopathy (left ventricular ejection fraction of 37%) is reported. Since coronary angiography could not identify a target lesion, secondary prophylactic implantable cardioverter-defibrillator (ICD) implantation was indicated. In light of slightly prolonged atrioventricular (AV) conduction (PQ 210 ms), narrow QRS (110 ms) and an elevated stroke risk (CHA_2_DS_2_-VASc score: 6), the decision was taken to implant a VDD ICD system (Intica 5 VR‑T DX, Biotronik SE & Co., Berlin, Germany) with the capability of monitoring atrial arrhythmias.

## Therapy and its course

**Fig. 1:**
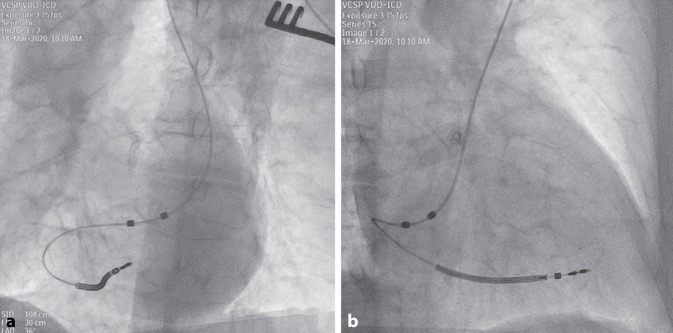
Left (**a**) and right (**b**) anterior oblique fluoroscopy projections of a VDD implantable cardioverter-defibrillator lead inserted via a persistent left superior vena cava

**Fig. 2:**
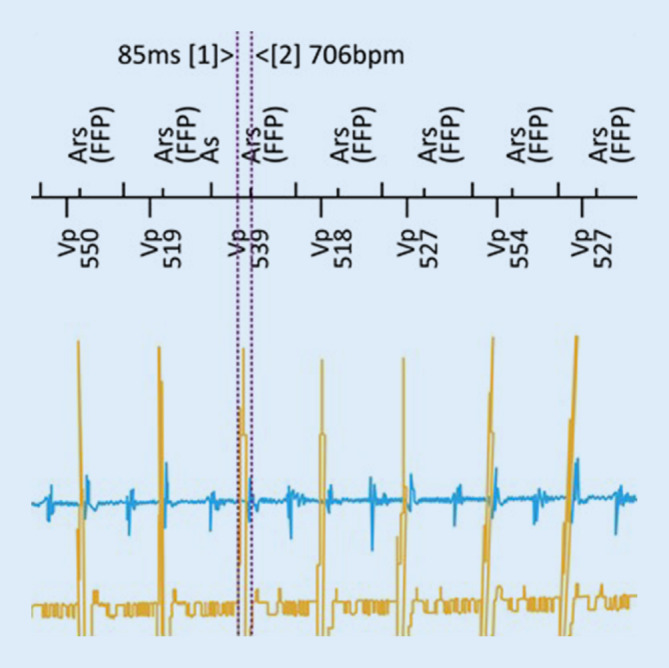
A double potential typical for coronary sinus was recorded in the atrial channel during atrioventricular-sequential ventricular pacing, which was successfully blanked out with the prolongation of the far-field protection window (*FFP*)

**Fig. 3:**
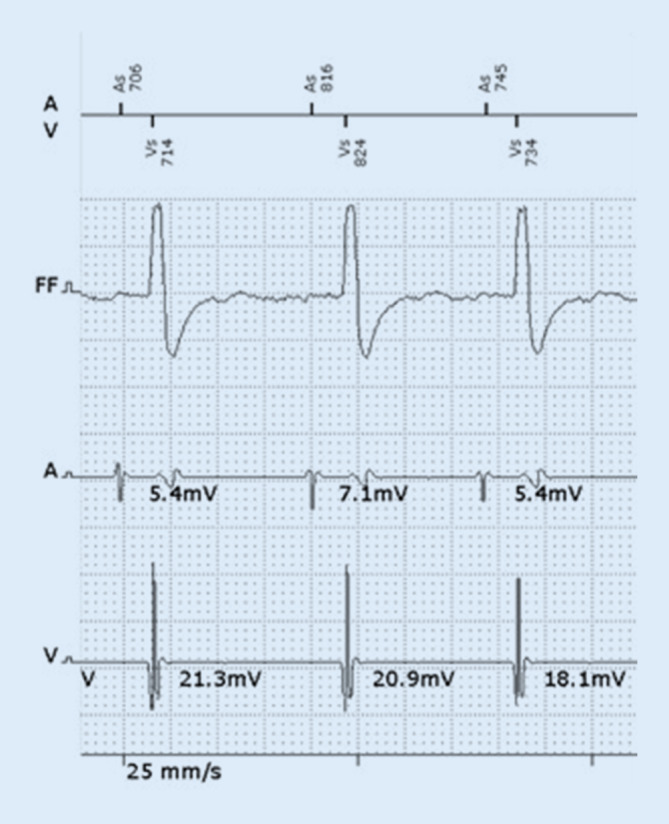
Sensing test at 1‑year follow-up showing high and stable atrial and ventricular signal amplitudes without any far-field oversensing

After starting the procedure from a typical left infraclavicular side, a persistent left superior vena cava (LSVC) was recognized and no relevant left to right venous communication could be identified by intraoperative venography. Hence, the lead (Plexa ProMRI DF‑1 DX 65/15, Biotronik SE & Co., Berlin, Germany) was successfully placed from the left subclavian approach via the LSVC by using a hand-shaped stylet (Fig. 1). Beside a high atrial sensing amplitude (8.7 mV), a double potential typical for coronary sinus was recorded directly after implantation in the atrial channel, especially during AV-synchronous ventricular pacing, as expected (Fig. 2). The far-field signal could be successfully blanked out with prolongation of the far-field protection window. Appropriate device function with stable and high atrial sensing amplitude was demonstrated at follow-up (4.3–6.5 mV at 1 year) (Fig. 3).

## Discussion

To preserve the benefit of atrial sensing without the need to implant an additional lead, a single-lead ICD system with a floating atrial dipole (VDD or DX ICD) has been developed [1]. The VDD ICD system offers an additional atrial intracardiac electrogram, with early detection of atrial arrhythmias, possibly improved supraventricular tachycardia discrimination and AV-sequential pacing in single-lead devices. Moreover, it can be upgraded to a two-lead cardiac resynchronization therapy (CRT)-DX system in the case of stable, long-term atrial sensing and a developing need for CRT. The feasibility of the VDD ICD system in the case of LSCV may be of particular interest.

The incidence of a persistent LSVC has been reported in up to 0.66% of ICD recipients [2]. Lead placement may be technically challenging, especially in the case of absent right superior vena cava. Although most cases can be accomplished with a reliable outcome [2], in some patients implantation of alternative systems should be considered [3, 4].

The current case represents an optimal candidate for a VDD ICD: slightly prolonged AV conduction with a potential need for AV-synchronous pacing and/or upgrade to CRT in the future and a greatly elevated risk of stroke [1]. However, implantation of more complex leads—such as an ICD lead—is often technically challenging in patients with cardiac anatomic variants. To the best of the authorsʼ knowledge, this is the first report demonstrating the feasibility of the VDD ICD system in the case of LSCV.

## Declarations

For this article no studies with human participants or animals were performed by any of the authors. All studies performed were in accordance with the ethical standards indicated in each case. Additional written informed consent was obtained from all individual participants or their legal representatives for whom identifying information is included in this article.
